# Antimicrobial Resistance and Genomic Characterization of Four *mcr*-1-Harbouring Foodborne *Salmonella* Isolates Recovered from Poultry in Saudi Arabia

**DOI:** 10.3390/antibiotics15060545

**Published:** 2026-05-29

**Authors:** Amani T. Alsufyani, Ashwaq Alhamed, Norah M. Alotaibi, Elaf Alshdokhi, Manal Almusa, Saleh Alaqeel, Hatim Almutairi, James J. Davis, Fahad Alreshoodi, Abdulrhman Alsawji, Majed F. Alghoribi, Sulaiman M. Alajel, Lenah E. Mukhtar

**Affiliations:** 1 Saudi Food and Drug Authority (SFDA), Riyadh 11671, Saudi Arabia; atsufyani@sfda.gov.sa (A.T.A.); as.hamed@sfda.gov.sa (A.A.); nmaotaibi@sfda.gov.sa (N.M.A.); eashdokhi@sfda.gov.sa (E.A.); mamusa@sfda.gov.sa (M.A.); siakeel@sfda.gov.sa (S.A.); hatim.almutairi@hotmail.com (H.A.); fmreshoodi@sfda.gov.sa (F.A.); sulaimanalajel@gmail.com (S.M.A.); 2 Consortium for Advanced Science and Engineering, University of Chicago, Chicago, IL 60637, USA; jjdavis@anl.gov; 3 Division of Data Science and Learning, Argonne National Laboratory, Argonne, IL 60439, USA; 4 Infectious Disease Research Department, King Abdullah International Medical Research Center, Riyadh 14611, Saudi Arabia; alswajiab@gmail.com (A.A.); alghoribima@gmail.com (M.F.A.); 5 Department of Basic Science, College of Science and Health Professions, King Saud bin Abdulaziz University for Health Sciences, Riyadh 14611, Saudi Arabia

**Keywords:** *Salmonella* enterica, *mcr*-1 gene, antimicrobial resistance, whole genome sequencing

## Abstract

**Background/Objectives**: The emergence of colistin resistance mediated by the *mcr*-1 gene in *Salmonella* enterica poses a significant public health concern. In 2022, *mcr*-1 was identified for the first time in *Salmonella* isolates recovered from chicken meat in Saudi Arabia, prompting the need for further genomic investigation. **Methods**: Four *Salmonella* enterica isolates—two serovar *Minnesota* and two serovar *Infantis*—underwent antimicrobial susceptibility testing (AST), whole-genome sequencing (WGS), and bioinformatics analyses including antimicrobial resistance genes (ARGs), virulence factors, plasmid replicons, and *mcr*-1 locations. **Results**: All isolates exhibited resistance to colistin, polymyxin B, and multiple first-line antibiotics. All four isolates were classified as multidrug-resistant (MDR). The *mcr*-1 gene was plasmid-borne in all isolates, located on IncI1_1_Alpha plasmids in *S. Minnesota* and IncFIB(K)_1_Kpn3 plasmids in *S. Infantis*. Additional antimicrobial resistance genes (ARGs) were detected, including *bla_CTX-M-65_*, *qnrB5*, *ermB*, and aminoglycoside-modifying enzymes, alongside multiple efflux pump genes. Virulence gene profiles showed minor differences between serovars, including the presence of the *cdtB* toxin gene in *S. Infantis* isolates. Phylogenetic analysis indicated that the isolates clustered within a distinct clade, suggesting a potential common local source or clonal expansion. **Conclusions**: This study provides the first detailed genomic insight into *mcr*-1-positive *Salmonella* isolates from food in Saudi Arabia. The co-existence of resistance and virulence determinants, together with mobile plasmids carrying the *mcr*-1 gene, emphasizes the risk of dissemination through the food chain. These findings highlight the urgent need for integrated genomic surveillance and strengthened antimicrobial stewardship within a One Health framework.

## 1. Introduction

Food-borne pathogens have significant impacts on both human health and the economy [1]. Consequently, they are a concern for public health authorities as well as for the food and dairy industries [1]. According to the United States Food and Drug Administration (FDA), approximately 48 million cases of food-borne illnesses are officially reported each year [2]. These occurrences lead to an estimated 128,000 hospitalizations and 3000 deaths annually [2]. Food-borne pathogens include bacteria, viruses, parasites, and fungi; among these, bacterial infections are the most frequently associated with food-borne diseases [1,3].

In 2018, the Saudi Ministry of Health (MOH) reported 2191 cases of illness and 11 fatalities in Saudi Arabia attributed to food-borne bacterial infections. The highest percentage of bacteria reported in the annual report was *Salmonella* spp., accounting for 47.46% of the cases [4]. *Salmonella* is a major cause of foodborne diseases and a global public health concern [5]. It is a Gram-negative, rod-shaped, facultative anaerobic bacterium belonging to the family Enterobacteriaceae [5]. Globally, over 2600 serotypes have been identified, many of which are associated with enteric and systemic diseases in both domestic animals and humans [5]. *Salmonella* species are particularly prevalent in many food sources, especially poultry, which represent a major reservoir [6,7]. Indeed, poultry products have been described as the main vehicles for transmitting human salmonellosis, accounting for most foodborne outbreaks [6,7]. Chicken meat, the most widely consumed poultry product, is considered a key source of human salmonellosis [8]. In fact, the Centers for Disease Control and Prevention (CDC) estimates that 1 in every 25 packages of chicken in grocery stores is contaminated with *Salmonella* [9].

The primary antimicrobials commonly used for treatment include β-lactams, third- and fourth-generation cephalosporins, carbapenems, and tigecycline [10,11]. The extensive and often unregulated use of these antibiotics in both veterinary and human medicine has facilitated the emergence and dissemination of resistant strains [11]. Beyond its prevalence in poultry, *Salmonella* has become a serious concern due to its increasing resistance to essential antibiotics used in human medicine [12]. This resistance can lead to treatment failures, particularly when first-line therapies become ineffective, thereby necessitating the use of second-line or last-resort antibiotics such as carbapenems or polymyxins.

Colistin is recognized as the last resort treatment for multidrug-resistant *Salmonella* [13]. Colistin, also known as polymyxin E, is widely used in both animal and human healthcare [14]. Its antibacterial activity targets the bacterial cell membrane, specifically the lipopolysaccharide (LPS) component of the outer membrane [14]. In recent years, resistance to colistin has been reported in *Salmonella* isolates from both human and animal sources, raising significant One Health concerns [15]. The extensive use of colistin in food-producing animals, especially chicken, over the last decades has applied considerable selective pressure, resulting in the emergence of colistin-resistant strains of bacteria, including *Salmonella* [14,15]. This resistance is primarily due to the acquisition of mobilized colistin resistance (*mcr*) genes, which are frequently located on plasmids and can be horizontally transferred among bacterial populations [14]. Currently, ten plasmid-mediated *mcr* genes (*mcr*-1 to *mcr*-10) have been documented in the literature, conferring colistin resistance across several Enterobacteriaceae species [16,17]. Although the majority of *mcr* genes have been predominantly identified in *Escherichia coli*, several variants, including *mcr*-1 to *mcr*-9, have also been observed in *Salmonella* isolates [16,18,19]. Colistin resistance in *Salmonella* can also result from chromosomal mutations [20]. These mutations typically occur in regulatory systems such as *pmr*A/*pmr*B, *pho*P/*pho*Q, and *mgr*B, which alter the structure of lipopolysaccharide (LPS) and reduce colistin binding efficiency [20,21].

Globally, colistin resistance in *Salmonella* has been reported across various regions. Studies conducted in Asia, Europe, and North America have identified colistin-resistant *Salmonella* in food, animals, and clinical isolates, with prevalence rates ranging from 0.03% to over 17% [16,21,22]. Animal reservoirs, specifically poultry, significantly contribute to this spread. For instance, a study conducted in Bangladesh revealed a 92.68% colistin resistance rate among *Salmonella* strains isolated from chickens, with the *mcr*-1 gene being identified, underscoring the significance of poultry as a primary reservoir [23].

Despite advancements in understanding the genomic characteristics of *Salmonella enterica* from food, genomic data remain limited in Saudi Arabia. In particular, information regarding the genetic background of *mcr*-1-positive foodborne *Salmonella* isolates is scarce. This includes data on the structure of resistance plasmids, associated antimicrobial resistance genes (ARGs), and the potential for resistance dissemination through the poultry production chain. Our goal was to identify ARGs and characterize plasmids to elucidate the genomic basis of colistin resistance, verify the structure and position of *mcr*-1-bearing elements, and assess their potential role in resistance dissemination. This research offers critical molecular insights into colistin resistance in *Salmonella* derived from a major food source in Saudi Arabia.

## 2. Results

### 2.1. Genomic Serotyping and Multilocus Sequence Typing (MLST)

Four *Salmonella enterica* isolates were analyzed, belonging to two serovars: serovar *Infantis* (ST32) and serovar *Minnesota* (ST548). Serotyping results showed that two isolates belonged to serovar *Infantis* (clade C1) and shared identical antigenic profiles. In contrast, the other two isolates were identified as serovar *Minnesota* (clade L) and exhibited distinct antigenic profiles.

The multilocus sequence typing (MLST) analysis showed the following allelic profiles for the *Infantis* isolates: *aro*C(17), *dna*N(18), *hem*D(22), *his*D(17), *pur*E(5), *suc*A(21), and *thr*A(19). Conversely, the *Salmonella Minnesota* isolates had the following allelic profiles: *aro*C(13), *dna*N(11), *hem*D(25), *hisD*(197), *pur*E(12), *suc*A(71), and *thr*A(4).

### 2.2. Phenotypic Characterization of Antimicrobial Resistance

All isolates were tested against the fluoroquinolone class, including nalidixic acid and ciprofloxacin. Both *S. Infantis* isolates (SA43930 and SA43968) were resistant to nalidixic acid, whereas the *S. Minnesota* isolates (SA49317 and SA49319) were susceptible. For ciprofloxacin, three isolates (SA49317, SA49319, and SA43930) showed intermediate susceptibility, while SA43968 was resistant.

The aminoglycoside class included amikacin, streptomycin, and gentamicin, and all isolates exhibited resistance to these three antibiotics. Within the β-lactam class, all isolates were resistant to ampicillin (a penicillin-type antibiotic). In contrast, all isolates were susceptible to the carbapenems, including imipenem, meropenem, and ertapenem.

Cephalosporins tested included cefoxitin, cefotaxime, ceftazidime, ceftriaxone, ceftiofur, and cefepime. All isolates were resistant to cefoxitin and susceptible to cefepime. Among the *S. Minnesota* isolates, SA49319 was susceptible to cefotaxime, ceftazidime, and ceftriaxone, whereas SA49317 was resistant to cefotaxime and ceftriaxone and showed intermediate susceptibility to ceftazidime. Among the *S. Infantis* isolates, SA43930 was susceptible to ceftazidime but resistant to cefotaxime, ceftiofur, and ceftriaxone. Similarly, SA43968 showed intermediate susceptibility to ceftazidime and resistance to cefotaxime, ceftiofur, and ceftriaxone.

Both *S. Minnesota* isolates were resistant to azithromycin, whereas the *S. Infantis* isolates were susceptible. All four isolates were resistant to chloramphenicol, except SA49319, which remained susceptible. Within the sulfonamide class, sulfisoxazole and the combination antibiotic trimethoprim–sulfamethoxazole were tested. Resistance to sulfisoxazole was observed in three isolates (SA49317, SA49319, and SA43968), while SA43930 remained susceptible. Similarly, resistance to trimethoprim–sulfamethoxazole was detected in three isolates (SA49317, SA43930, and SA43968), whereas SA49319 was susceptible.

Importantly, all isolates exhibited resistance to colistin and polymyxin B. Temocillin was also tested and showed resistance in three isolates, while *S. Infantis* SA43930 remained susceptible. Aztreonam, a monobactam β-lactam antibiotic, was tested and found to be ineffective against all isolates.

Due to the observed resistance to aztreonam, cefotaxime and ceftazidime antibiotics, which is a typical indicator of Extended-Spectrum Beta-Lactamase (ESBL) activity, the phenotypic ESBL detection was conducted by evaluating cefotaxime and ceftazidime in combination with clavulanic acid. Not all isolates were tested positive for ESBL production (Table 1).

### 2.3. Multidrug Resistance Degree

Based on the antimicrobial susceptibility results presented in Table 1, the resistance profiles of the isolates were classified according to the internationally accepted definitions proposed by Magiorakos et al. (2012) [24]. According to these criteria, isolates are categorized as multidrug-resistant (MDR) when they exhibit non-susceptibility to at least one antimicrobial agent in three or more antimicrobial classes. All four isolates were classified as MDR. The isolates exhibited resistance to at least one antibiotic in multiple antimicrobial classes, including aminoglycosides, polymyxins, penicillins, monobactams, tetracyclines, phenicols, and folate pathway inhibitors. In addition, resistance to several cephalosporins was observed among the isolates. Despite this multidrug resistance profile, all isolates remained susceptible to several antimicrobial classes, particularly carbapenems (imipenem, meropenem, and ertapenem), glycylcyclines (tigecycline), and the fourth-generation cephalosporin cefepime. Susceptibility to additional antimicrobial agents varied among isolates, including azithromycin and selected cephalosporins.

Because susceptibility remained to multiple antimicrobial categories, none of the isolates met the criteria for extensively drug-resistant (XDR) classification, which requires susceptibility to only one or two antimicrobial classes. The resistance classification of the isolates is summarized in Table 2.

### 2.4. MAR Index

The isolates *S. Infantis* SA43968 and *S. Minnesota* SA49317 exhibited the highest Multiple Antibiotic Resistance (MAR) index values, both reaching 0.7. These values correspond to resistance to 18 and 17 antibiotics, respectively, across nine antimicrobial classes, out of a total of 26 antibiotics tested. The remaining isolates also showed MAR index values above 0.2 (Table 2).

### 2.5. Antimicrobial Resistance Genes (ARGs)

**Figure 1:**
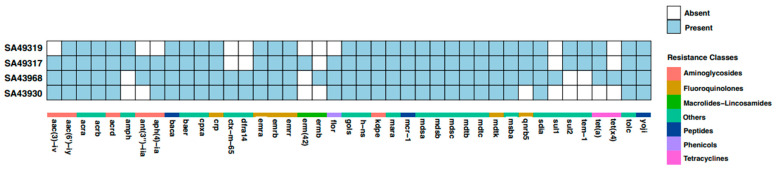
Heatmap displaying the presence or absence of antimicrobial resistance genes in four *Salmonella* enterica isolates. Each tile represents gene presence (blue) or absence (white). Genes are grouped by antimicrobial class, as indicated by color-coded categories. Antibiotic classes were assigned based on the CARD (Comprehensive Antibiotic Resistance Database) ontology.

The analysis of ARGs in the four hybrid fasta genome assemblies (SA43930, SA43968, SA49317, and SA49319) revealed a diverse and extensive array of resistance determinants. Figure 1 illustrates the identified ARGs across these samples, highlighting both shared and unique profiles. Several core genes were detected across all isolates, including efflux pump-associated genes (*mdsC*, *mdsB*, *mdsA*, *acrB*, *tolC*, *marA*, *kdpE*, *hns*, *sdiA*, *mdtK*, *mdtB*, *mdtC*, *baeR*, and *yojI*), aminoglycoside resistance genes (*aac(6′)-Iy*, *aph(4)-Ia*, and *aac(3)-IV*), and the colistin resistance gene *mcr*-1. In addition, peptide resistance-associated genes such as *bacA* and *yojI* were consistently present in all isolates. A β-lactam resistance gene, *bla_CTX-M-65_*, associated with extended-spectrum β-lactamase (ESBL) activity, was also detected in both *S. Infantis* isolates (SA43930 and SA43968). Distinct differences were observed among individual isolates. The *S. Infantis* isolate SA43930 uniquely harbored *acrA* and *acrD*, along with the mobile trimethoprim resistance gene *dfrA14*. In contrast, the *S. Infantis* isolate SA43968 contained an expanded set of ARGs, including *sul1*, *tet(A)*, *tet(X4)*, *ermB*, *and qnrB5*, contributing to a broader resistance profile. The *S. Minnesota* isolates SA49317 and SA49319 displayed highly similar ARG profiles. Both isolates carried genes such as *ampH*, *erm(42)*, *sul2*, and *tem-1*. Additionally, the quinolone resistance gene *qnrB5* was detected in these isolates, further contributing to their resistance repertoire (Figure 1).

### 2.6. Virulence Factor Genes

**Figure 2:**
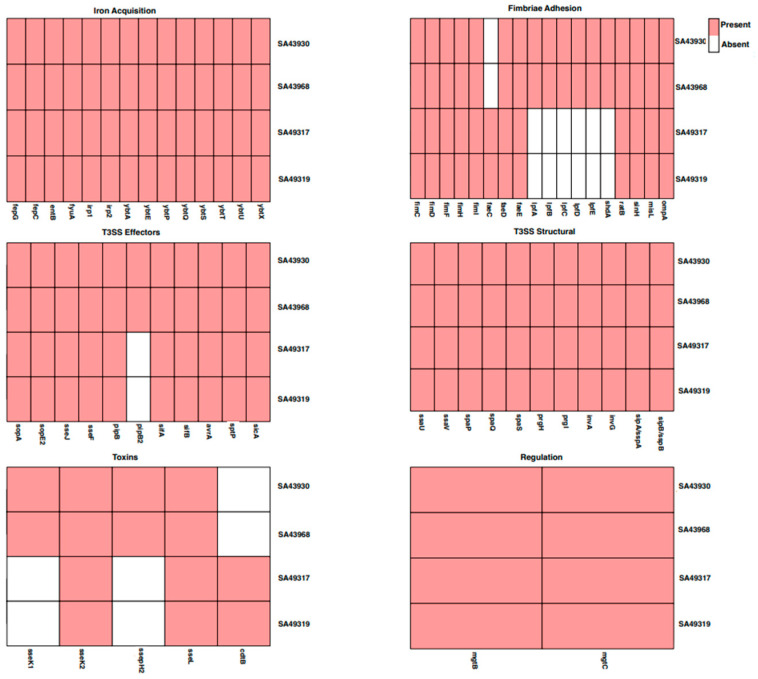
Heatmap showing the presence or absence of virulence genes in four *Salmonella* enterica isolates. Columns represent virulence genes grouped by molecular function (e.g., fimbriae, toxins, iron acquisition systems, Type III secretion system (T3SS) effectors and structural components, and regulatory elements). Rows represent individual isolates. Red indicates presence; white indicates absence. Both rows and columns were clustered to highlight patterns in virulence gene distribution.

The analysis of virulence-associated genes across the four *Salmonella* isolates revealed a diverse distribution of 117 identified factors. All isolates harboured multiple virulence genes associated with key pathogenic functions, including adhesion, invasion, intracellular survival, and secretion systems. These functional groups corresponded to several virulence categories, including fimbrial adhesion, toxins, iron acquisition systems, Type III secretion system (T3SS) effectors and structural components, as well as regulatory elements (Figure 2).

Most virulence genes, particularly those related to iron acquisition systems, T3SS structural components, and regulatory elements, were conserved across all isolates. However, variations were observed in specific virulence categories. Differences were most evident in genes associated with fimbrial adhesion and toxin production. The toxin gene *cdtB* was detected only in the *S. Infantis* isolates (SA43930 and SA43968). In contrast, several fimbrial genes were absent in the *S. Minnesota* isolates (SA49317 and SA49319). Overall, the virulence gene profiles were largely conserved among the isolates, with only minor variations distinguishing the two serovars (Figure 2).

### 2.7. Plasmid Replicon Profiles

**Figure 3:**
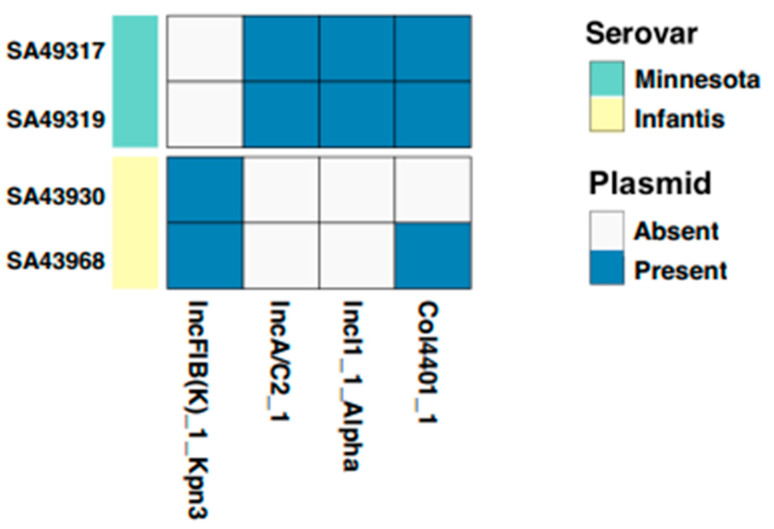
Heatmap displaying the presence or absence of plasmid replicons in four *Salmonella* enterica isolates. Columns represent plasmid replicons and rows correspond to individual isolates. Each tile represents the presence (blue) or absence (white) of plasmids. Columns are grouped by serovars: the yellow reflect the *Salmonella* serovar *Infantis*, and the green reflect the *Salmonella* serovar *Minnesota*.

The *S. Infantis* isolate SA43930 carried a single plasmid replicon type, IncFIB(K)_1_Kpn3, showing a relatively simple plasmid composition. In contrast, the *S. Infantis* isolate SA43968 harbored two plasmid replicons, IncFIB(K)_1_Kpn3 and Col440I_1. The *S. Minnesota* isolates SA49317 and SA49319 exhibited identical plasmid replicon profiles, each containing three plasmid types: IncA/C2_1, IncI1_1_Alpha, and Col440I_1 (Figure 3).

### 2.8. MCR-1 Location in Plasmids

**Figure 4:**
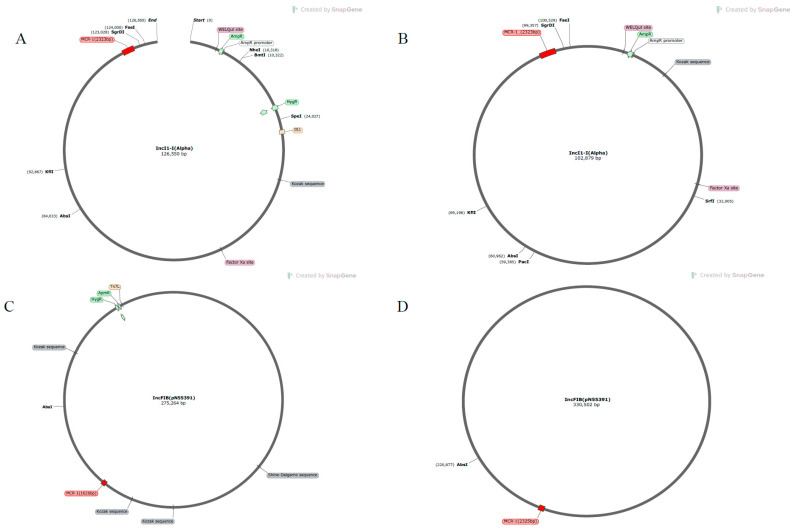
A figure shows genomic loci of the *mcr*-1 gene in four *Salmonella* isolates (**A**–**D**). Panels (**A**) (S49317) and (**B**) (S49319) represent *S. Minnesota* isolates, in which *mcr*-1 is located on IncI1_1_Alpha plasmids. Panels (**C**) (S43930) and (**D**) (S43968) correspond to *S. Infantis* isolates, where *mcr*-1 gene is carried on IncFIB(pN55391) plasmids.

In all four *Salmonella* enterica isolates analyzed, the *mcr*-1 gene was located on plasmids; however, the plasmid replicon types differed by serovar. In the two *S. Minnesota* isolates, the *mcr*-1 gene was carried on IncI1_1_Alpha plasmids. In contrast, the *mcr*-1 in the two *S. Infantis* isolates was located on IncFIB (pN55391)-type plasmids (Figure 4).

### 2.9. Pangenome Analysis

**Figure 5:**
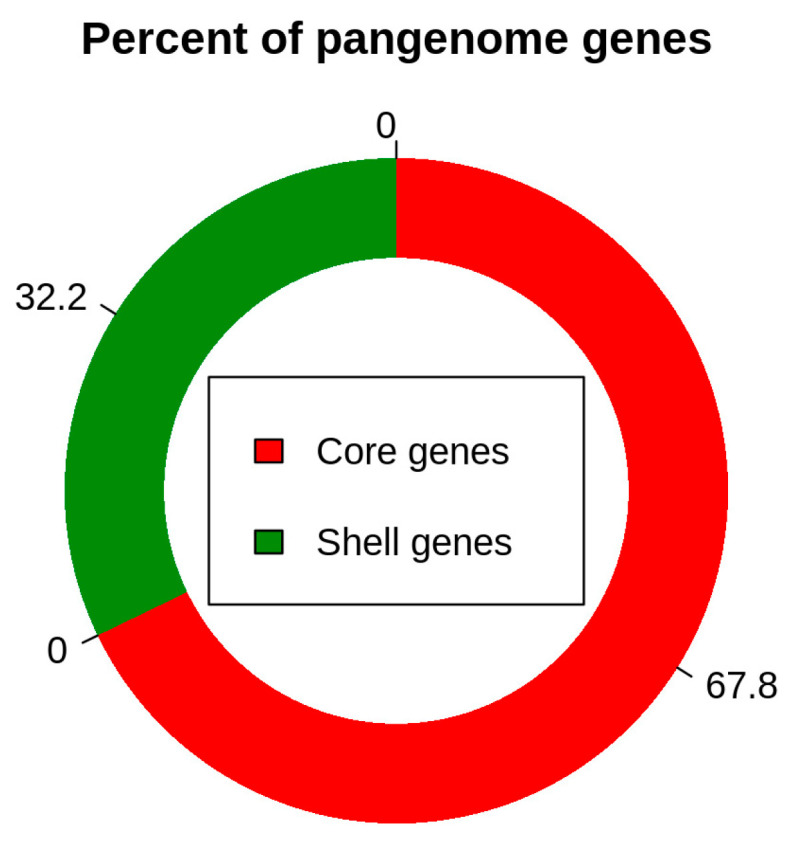
Pangenome composition of the four *Salmonella enterica* isolates. The chart shows the percentage of genes classified as core genes (red, 67.8%) and shell genes (green, 32.2%). No soft-core or cloud genes were detected in the dataset.

Pangenome analysis showed that 67.8% of the genes were core genes, shared across all isolates, while 32.2% were classified as shell genes. No soft-core or cloud genes were detected in this dataset (Figure 5).

### 2.10. Phylogenetic Tree of MCR Proteins

**Figure 6:**
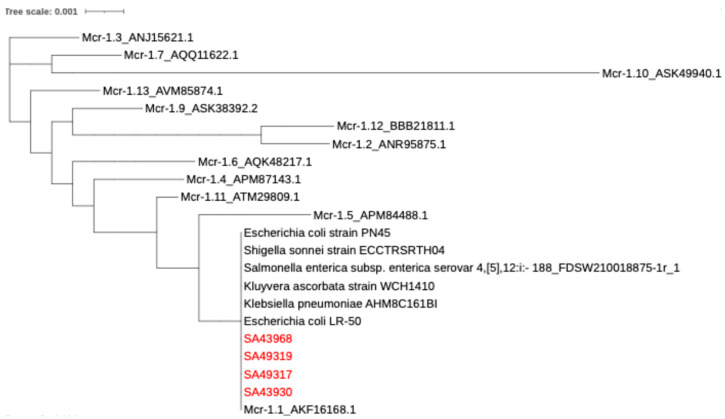
Phylogenetic tree of MCR proteins. MCR proteins from the four *Salmonella* enterica strains sequenced in this study are highlighted in red. For comparative context, representative MCR proteins from GenBank (with accession numbers) and MCR-1.1 proteins from BV-BRC are also included.

A phylogenetic tree was constructed to assess the genetic relatedness of *Salmonella* isolates carrying the *mcr* gene, using publicly available *Salmonella* genomes from global sources (GenBank). The four isolates formed a closely related clade, suggesting a shared origin. Conversely, the boarder tree topology revealed substantial genetic diversity among the global isolates (Figure 6).

## 3. Discussion

The *mcr*-1 gene was first reported in *Salmonella enterica* isolates recovered from chicken meat samples in Saudi Arabia in 2022 [25]. Consequently, four *Salmonella enterica* isolates, including two strains of *Minnesota* and two strains of *Infantis*, were further analyzed and subjected to comprehensive genomic investigation to better understand their resistance mechanisms, virulence potential, and plasmid structure, which are critical factors for assessing the risk of potential dissemination within the food production environment.

In Saudi Arabia, several reviews have highlighted the growing concern of colistin resistance in Gram-negative bacteria, excluding *Salmonella*, particularly in clinical settings. However, few studies have specifically investigated *Salmonella enterica* from food sources. In 2022, it was reported that two *Salmonella Minnesota* strains harbouring the *mcr-1.1* gene were isolated from chicken meat in Riyadh city in Saudi Arabia [18]. These findings are consistent with the increasing global dissemination of *mcr*-harbouring *Salmonella* reported worldwide. Public database investigations identified 2279 *mcr*-positive *Salmonella* genomes across 37 countries and five continents [18]. The majority of these isolates were sourced from human samples (39.5%), followed by food (32.6%), animals (13.7%), and the environment (4.4%) [18]. The study showed that *mcr*-9.1 was the most common variant (65.2%), followed by *mcr-1.1* (24.4%) [18]. These findings highlight the widespread distribution and ecological diversity of *mcr*-harboring *Salmonella*, supporting the notion that food-producing animals and food products represent important reservoirs within a global One Health framework.

The increasing prevalence of multidrug-resistant *Salmonella* strains in poultry poses a significant public health concern, as these bacteria can transmit ARGs to other bacteria and cause challenging, difficult-to-treat infections [26]. Thus, understanding their genomic characteristics is essential for developing targeted interventions to mitigate the impact of multidrug-resistant *Salmonella* on both animal and human health.

The analysis of ARGs across samples revealed a complex network of resistance mechanisms, including enzymatic modification of antibiotics and efflux pump-mediated drug expulsion. Efflux pumps are essential for conferring resistance to a wide range of antibiotics as they actively remove a broad range of antibiotics from the bacterial cell, lowering intracellular concentrations and preventing the drugs from reaching their target, thus, enabling bacteria to survive under the stress of antibiotics [27,28]. Similar efflux-associated resistance profiles have been widely reported in *Salmonella enterica*, particularly in poultry-associated lineages such as ST32, where genes like *acrB* and *tolC* play a central role in multidrug resistance phenotypes [29]. In this study, key efflux pump genes, including *mdsC*, *mdsB*, *mdsA*, *acrB*, *tolC*, *marA*, *kdpE*, *H-NS*, *sdiA*, *mdtK*, *mdtB*, *mdtC*, *baeR*, and *yojI* were identified. Many of these are implicated in resistance to β-lactams, aminoglycosides, and fluoroquinolones [30]. The presence of these regulators and structural components suggests a coordinated regulatory network contributing to adaptive resistance under antimicrobial pressure.

In addition to efflux mechanisms, enzymatic resistance such as β-lactamase production (*bla_CTX-M-65_*) and aminoglycoside-modifying enzymes (*aac(6′)-Iy*) enables bacteria to chemically inactivate antibiotics [31,32]. β-lactamases hydrolyze β-lactam antibiotics (e.g., penicillins and cephalosporins), while aminoglycoside-modifying enzymes deactivate aminoglycosides, rendering them ineffective [33]. The detection of *bla_CTX-M-65_*, in particular, aligns with previous global reports describing its association with epidemic *Salmonella Infantis* clones linked to poultry production chains [34]. The presence of these genes in the investigated samples indicates a significant challenge in treating infections caused by these bacteria, especially as resistance can evolve and disseminate rapidly, rendering many first-line treatments ineffective [34].

The diversity of resistance profiles observed among the samples suggests a multifaceted resistance architecture, enabling the bacteria to evade multiple antibiotic classes. Notably, resistance to the last-resort antibiotic colistin, mediated by the *mcr*-1 gene, poses a critical public health concern due to its potential for rapid dissemination through mobile genetic elements [35]. According to Sun et al., 2023, the emergence of the *mcr*-1 gene, which confers resistance to colistin, has further complicated efforts to control resistant pathogens [27]. Colistin, a last-resort antibiotic for carbapenem-resistant infections (e.g., *Klebsiella pneumoniae*, *Escherichia coli*), is now at risk due to the spread of *mcr*-1 via mobile plasmids [36,37]. The ability of this gene to transfer horizontally among bacterial species magnifies the risk of resistance in both clinical and agricultural settings, undermining one of the last effective treatment options [27].

The detection of *mcr*-1 in both clinical and non-clinical settings, including animals, food, and the environment, highlights a potential risk of dissemination through the food chain rather than direct evidence of transmission to humans [27,28]. This distinction is important, as the current study does not include human isolates or epidemiological linkage data. Therefore, while these findings suggest that poultry-associated *Salmonella* may act as a reservoir of colistin resistance, direct transmission pathways cannot be confirmed and require further investigation [28]. This growing complexity of resistance mechanisms underscores the urgent need for continuous surveillance of ARG profiles to monitor the emergence of new strains, especially those conferring resistance to multiple antimicrobial classes. Additionally, the findings of this study further highlight the need for novel therapies and rational antimicrobial use in healthcare and agriculture, where inappropriate antibiotic practices can drive resistance evolution.

Comparative analysis of virulence genes revealed distinctions between the *S. Infantis* and *S. Minnesota* strains, despite a shared genetic background, which could affect their pathogenicity and therapeutic efficacy. The *S. Minnesota* strains possessed 117 virulence genes, while the *S. Infantis* strains carried 110. These findings are broadly consistent with previous genomic studies indicating that *S. Infantis* often carries enhanced virulence determinants linked to host adaptation and persistence [38,39,40,41,42]. Interestingly, the cdtB gene (encoding cytolethal distending toxin B) was detected exclusively in the *S. Infantis* strains and is known to induce host DNA damage, cell cycle arrest, and apoptosis [41]. Additionally, the *S. Infantis* strains harboured a complete set of long polar fimbriae (lpf) genes, enhancing their ability to adhere to and colonize host tissues [42]. Moreover, both *S. Infantis* strains contained immune evasion genes (*sspH2* and *sseK1*), which modulate host cell signaling and suppress inflammatory responses [38,39]. These genes were also detected in the *S. Minnesota strains*. Furthermore, all isolates carried the *pipB2* gene, which facilitates intracellular survival by manipulating host cell trafficking and promoting bacterial persistence within host cells [40]. These virulence determinants are consistent with those reported in previous studies in *Salmonella enterica*, particularly poultry-associated lineages, where enhanced colonization and persistence are key features of pathogenicity. Together, these molecular features highlight the complex relationship between antimicrobial resistance and virulence and suggest potential serovar-specific differences in pathogenic strategies between *S. Infantis* and *S. Minnesota*.

Plasmid profiling revealed significant diversity in plasmid replicon content among the *Salmonella* strains. The *S. Infantis* SA43930 strain harboured a single plasmid replicon, IncFIB(K)_1_Kpn3, which is commonly associated with virulence and extended-spectrum β-lactamase (ESBL) genes in Enterobacteriaceae [36]. The presence of a single plasmid replicon may reflect a more streamlined genetic architecture, potentially limiting the strain’s capacity for horizontal gene transfer [37]. In contrast, *S. Infantis* SA43968 carried two plasmid replicon types, IncFIB(K)_1_Kpn3 and Col440I_1, which have been previously linked to resistance and virulence factors, suggesting greater adaptability and genomic flexibility [43,44]. The *S. Minnesota* strains SA49317 and SA49319 shared similar plasmid profiles, each harboring three plasmid replicon types: IncA/C2_1, IncI1_1_Alpha, and Col440I_1, indicative of a possible common origin or evolutionary trajectory. Importantly, the plasmid types identified in this study (IncFIB, IncI1, IncA/C2) have been widely reported in association with antimicrobial resistance and horizontal gene transfer in Enterobacteriaceae, including *Salmonella* [36,37,45].

In the current study, of particular note is the detection of the IncA/C2 plasmid, which is well known to be associated with antimicrobial resistance genes in Enterobacteriaceae, including quinolone resistance genes (*qnrB1* and *qnrS1*), aminoglycoside resistance genes (*armA* and *rmtC*), and β-lactamase genes (*bla_CMY-2_* and *bla_CTX-M-15_*) [46]. These findings emphasize the role of plasmids in bacterial adaptation and the importance of plasmid surveillance to track resistance evolution.

The localization of *mcr*-1 on two distinct plasmid types, IncI1_1_Alpha in *S. Minnesota* strains and IncFIB(K)_1_Kpn3 in *S. Infantis* strains, raises concern about the mobility and environmental adaptability of colistin resistance determinants. IncI1_1_Alpha plasmids are known for their high conjugation efficiency and broad host range, enabling rapid horizontal gene transfer among *Enterobacteriaceae* [47]. In contrast, IncFIB(K)_1_Kpn3 plasmids are commonly associated with *S. Infantis* lineages adapted to avian hosts and often carry both resistance and virulence genes [48]. Similar associations between IncFIB(K)_1_Kpn3 plasmids, multidrug resistance, and virulence determinants have previously been reported in poultry-associated *S. Infantis* and *S. Minnesota* isolates from Saudi Arabia [49]. This dual plasmid context suggests both high mobility and ecological adaptation, increasing the potential for dissemination across bacterial populations.

The presence of *mcr*-1 on transmissible plasmids, even if rarely detected globally, represents a substantial threat due to its potential for widespread dissemination across pathogenic and commensal bacterial populations. This raises the risk of dissemination in the food environment and the gastrointestinal tracts of humans and animals. While colistin is not typically used to treat *Salmonella* infections, its application in livestock promotes selective pressure that facilitates the persistence of *mcr*-carrying strains. Furthermore, *mcr*-1 is frequently linked to plasmid types such as IncI2, IncHI2, and IncX4, which are known for their high transfer efficiency [28]. Compared to globally reported *mcr*-1-harboring *Salmonella*, the plasmid profiles observed here are partially consistent with previously described IncI2, IncHI2, and IncX4 backbones, although the presence of IncFIB-associated *mcr*-1 may indicate regional variation or emerging plasmid configurations [28].

Apart from the presence of the *mcr*-1 gene on transmissible plasmids, the resistance of these *Salmonella* isolates to first-line antibiotics, including third-generation cephalosporins, quinolones, and trimethoprim/sulfamethoxazole, raises serious public health concerns. Treatment failure, prolonged infection, and increased reliance on last-resort antibiotics such as carbapenems may result, further driving resistance selection, increasing healthcare costs, and prolonging hospital stays.

Although the isolates belonged to two distinct serovars and exhibited some genetic heterogeneity (as shown in Figure 5), they all carried the *mcr*-1 gene on plasmids. The presence of *mcr*-1 across genetically distinct isolates suggests that horizontal gene transfer may play a key role in its dissemination; however, high-resolution genomic approaches (e.g., SNP-based phylogeny or core genome analysis) would be required to confirm this hypothesis [50]. The presence of *mcr*-1-bearing plasmids in genetically similar yet serotypically different strains points to a common environmental or ecological reservoir of resistance. This reinforces the critical role of mobile genetic elements in resistance dissemination and highlights the need to integrate plasmid-level tracking into epidemiological surveillance and containment strategies [43].

We propose a potential indirect association between the *mdtB*, *mdtC*, and *mdtK* efflux pump genes and the *mcr*-1 gene. While this relationship remains speculative, it may reflect coordinated stress-response pathways involved in membrane adaptation and antimicrobial resistance. The *mcr*-1 gene modifies lipid A in the bacterial outer membrane, reducing the binding affinity of colistin [44], whereas the *mdt* genes encode efflux systems that expel antibiotics and toxic compounds, thereby lowering intracellular drug concentrations [51]. Although regulatory systems such as PmrA/PmrB and PhoP/PhoQ [52] were not identified among the detected resistance genes in these isolates, co-activation of *mdt* and *mcr* genes may occur under environmental stress conditions (e.g., low magnesium levels or antibiotic exposure), potentially through broader regulatory pathways controlling membrane modification and stress responses [53,54,55]. Such interactions, if confirmed, could indicate complementary resistance mechanisms enhancing bacterial survival under antimicrobial pressure; however, experimental validation is required to support this hypothesis.

This may suggest a complementary interaction that enhances resistance to antimicrobial peptides. Phylogenetic analysis showed that the four *Salmonella* isolates clustered together with previously reported *MCR-1* reference proteins included from public databases (Figure 6), suggesting that the identified *MCR* proteins are closely related to globally distributed *mcr*-1 variants rather than highly divergent forms. This finding supports the widespread dissemination of plasmid-mediated colistin resistance across different geographic regions and ecological settings. However, this observation should be interpreted with caution, as the analysis was based on limited genomic resolution. Protein-level or low-resolution phylogenetic approaches are insufficient to infer clonal expansion or epidemiological linkage, and higher-resolution methods (e.g., SNP-based phylogeny or core genome analysis) would be required to accurately determine relatedness and potential dissemination patterns.

Overall, this study provides important genomic insight into *mcr*-1–harboring *Salmonella enterica* from poultry in Saudi Arabia.

The findings suggest that poultry-associated *Salmonella* may represent a potential reservoir of colistin resistance genes within the food production system.

However, several limitations should be acknowledged, including the small sample size, the absence of human isolates, and the lack of epidemiological linkage or high-resolution genomic analysis, which limit the ability to infer transmission dynamics.

Future studies should incorporate larger datasets, include human and environmental isolates, and apply high-resolution genomic approaches to better understand the dissemination pathways of *mcr*-1 and associated resistance determinants within a One Health framework.

## 4. Materials and Methods

### 4.1. Sample Collection

The *Salmonella enterica* isolates analyzed in this study were obtained from a surveillance collection of chicken meat samples previously described by Huang et al. [56]. Briefly, samples were collected from poultry products obtained from supermarkets across Saudi Arabia as part of routine monitoring programs conducted at the Microbiology Reference Laboratory of the Saudi Food and Drug Authority (SFDA) between 2020 and 2022. Isolation and identification of *Salmonella* were performed according to ISO 6579-1:2017/Amd 1:2020 standards, as described previously [56,57,58]. For the present study, four isolates carrying the *mcr*-1 gene were selected from this collection, including two *S. Minnesota* isolates (SA49317 and SA49319) and two *S. Infantis* isolates (SA43930 and SA43968).

### 4.2. Identification of Colistin Resistance in Salmonella Isolates

#### 4.2.1. Antimicrobial Susceptibility Testing

Susceptibility testing of the *Salmonella* isolates was performed using the broth microdilution method with the Sensititre^®^ system (Thermo Fisher Scientific Microbiology, Oakwood Village, OH, USA) to determine the minimum inhibitory concentrations (MIC) of antibiotics. Testing panels included EUVSEC3, GNX3F, and CMV3GNF (Thermo Fisher Scientific, Waltham, MA, USA), selected based on the EU surveillance system. The panels covered nine major antibiotic classes: aminoglycosides; β-lactams (including penicillins, cephalosporins, carbapenems, monobactams, and semi-synthetic β-lactams); macrolides; tetracyclines (glycylcyclines); quinolones (fluoroquinolones); sulfonamides; polypeptides (polymyxins); β-lactamase inhibitors (β-lactam combination agents); and amphenicols.

Briefly, all isolates, *S. Minnesota* SA49317, *S. Minnesota* SA49319, *S. Infantis* SA43968, and *S. Infantis* SA43930, were incubated on nutrient agar plates for 24 h at 37 °C. A single colony was transferred from the agar plates into 5 mL of sterile demineralized water (Cat no. T3339, Thermo Fisher Scientific, Waltham, MA, USA) to prepare a suspension of approximately 10^7^–10^8^ colony-forming units (CFU)/mL (0.5 McFarland) using a Nephelometer (Sensititre™ Nephelometer, Thermo Fisher Scientific, Waltham, MA, USA). Then, 10 µL of the suspension was added to a vial with 11 mL of Mueller-Hinton broth (MHB) (Cat no. T3462, Thermo Fisher Scientific, Waltham, MA, USA), vortexed, and then 50 µL was dispensed into each plate well using the Sensititre AIM™ Automated Inoculation Delivery System (Thermo Fisher Scientific, Waltham, MA, USA). After inoculation, each plate was sealed with the provided adhesive film and incubated for 18–24 h at 37 °C. The *E. coli* strain ATCC 25922 was used as the quality control strain. The plates were then read using the Sensititre™ OptiRead™. MIC values for all antibiotics were interpreted according to the Clinical & Laboratory Standards Institute (CLSI) breakpoint criteria using Sensititre SWIN (SWIN™ Software System, V3.4), except for tigecycline, which was interpreted using the FDA Antibacterial Susceptibility Test Interpretive Criteria [59,60]. Further, for streptomycin, azithromycin, cefoxitin, nalidixic acid, and ceftiofur, *Salmonella* MIC interpretations were based on the National Antimicrobial Resistance Monitoring System (NARMS) guidelines [10].

#### 4.2.2. Multiple Antibiotic Resistance (MAR) Index

The MAR index, which is a proven and dependable approach for identifying the origin of resistant bacteria, was calculated [61]. This method is used to distinguish between bacteria that come from diverse origins by targeting antibiotics regularly used in human treatment [61,62].

The MAR index was calculated as a measure of antibiotic resistance risk, indicating the possible origin of contamination and the degree of antibiotic misuse [61,62]. This method provides a cost-effective alternative to genotypic characterization for bacterial source tracking. The MAR index was calculated using the formula [62]:

MAR index = a/b

where ‘a’ represents the number of antibiotics to which an isolate was resistant, and ‘b’ represents the total number of antibiotics evaluated for susceptibility testing.

#### 4.2.3. Detection of Resistance Degree

Antibiotic resistance profiles were classified according to CLSI, EUCAST, and FDA guidelines, as well as the consensus of expert groups for Enterobacteriaceae, including *Salmonella* [24]. According to CLSI, EUCAST, and FDA guidelines, together with the expert group consensus, the parameters used to determine the degrees of antibiotic resistance in Enterobacteriaceae, including *Salmonella*, categorize bacteria as multidrug-resistant (MDR), extensively drug-resistant (XDR), or pan drug-resistant (PDR) [24]. This classification is based on resistance to multiple antimicrobial classes, including aminoglycosides, penicillins, cephalosporins, carbapenems, fluoroquinolones, folate pathway inhibitors, monobactams, tetracyclines, polymyxins, phosphonic acids, and phenicols [24]. Based on resistance to these antimicrobial classes, isolates were categorized as follows [24]: (1) multidrug-resistant (MDR), defined as resistance to at least one agent in three or more antimicrobial classes; (2) extensively drug-resistant (XDR), defined as resistance to at least one agent in all but two or fewer antimicrobial classes; and (3) pan drug-resistant (PDR), defined as resistance to all agents in all antimicrobial classes tested. These definitions were used to categorize the resistance profiles of the isolates in this study.

### 4.3. Whole-Genome Sequencing (WGS)

Genomic DNA was extracted from four *Salmonella* isolates using the QIAamp DNA Mini kit (Qiagen, Valencia, CA, USA) according to the manufacturer’s instructions, facilitated by a semi-automated QIAcube machine. The purity of the extracted DNA was confirmed by measuring the A260/A280 ratio with QIAxpert (Qiagen, CA, USA), targeting a ratio of ≥1.8. DNA concentrations were quantified using a QFX Fluorometer (Denovix Inc., Wilmington, DE, USA) based on the manufacturer’s guidelines.

Library preparation was carried out using the Nextera XT DNA Library Prep Kit and the Nextera XT Index Kit (Illumina Inc., San Diego, CA, USA). Post-PCR products were purified using 45 μL of Agencourt AMPure XP magnetic beads (Beckman Coulter, Brea, CA, USA) at a 3:2 bead-to-sample ratio. Next, the libraries were normalized with Qubit quantification and pooled to a final concentration of 4 nM. Paired-end sequencing was then conducted on the Illumina MiSeq platform using a 600-cycle MiSeq v3 reagent kit with 5% PhiX control (Illumina Inc.). Adapter trimming and FASTQ file generation were performed automatically by the MiSeq system. Raw data were demultiplexed using Illumina’s bcl2fastq tool (version 2.20). The quality of the raw reads (FASTQ files) was assessed using FastQC v0.11.9 (4). Long-read assembly was performed with Flye for isolates SA43930, SA43968, SA49317, and SA49319. The long reads for this strain were generated on a MinION sequencer (Oxford Nanopore Technologies, Oxford, UK) using the ligation sequencing kit SQK-LSK109. Default parameters were used for all software unless otherwise specified. The MinION raw data were base-called using Guppy (version 4.3.4), which is integrated into MinKNOW (version 21.02.5), followed by filtering of reads with a minimum Q score of 7 and a minimum read length of 1000 bp.

### 4.4. Bioinformatics Analysis

Short reads were mapped to the long-read assemblies using BWA-MEM V0.7.17.2, with alignments saved in SAM format. SAM files were then converted, sorted, and indexed into BAM files using SAMtools V1.22. These steps ensured the data was prepared appropriately for downstream analysis. The consensus sequences were generated using SAMtools consensus, producing FASTA files: 43930_cons.fasta, 43968_cons.fasta, 49317_cons.fasta, and 49319_cons.fasta.

Then the assemblies were analyzed using the TORMES pipeline (v1.3). Low-quality reads and adapters were removed using Trimmomatic V0.40. Assembly quality was assessed via QUAST V5.2.0, and genome annotation was conducted using Prokka V1.15.6. Multi-locus sequence typing (MLST) was performed using the integrated mlst tool in TORMES.

In addition, within TORMES pipeline, the antibiotic resistance genes (ARGs) were identified via the Comprehensive Antibiotic Resistance Database (CARD), while virulence factors were detected using the VFDB database. PlasmidFinder V2.1 was used to identify plasmid replicons, indicating the presence of mobile genetic elements.

To identify and localize the *mcr*-1 gene, annotated genome assemblies from TORMES pipeline were imported into SnapGene (Insightful Science; snapgene.com). Using SnapGene’s advanced search, assemblies were screened for *mcr*-1, and identified gene regions were visually verified to map their exact positions within each genome.

### 4.5. Phylogenetic Tree Construction

Reference MCR protein sequences were obtained from GenBank [63], and additional homologous sequences were downloaded from the Bacterial and Viral Bioinformatics Resource Center (bv-brc.org) on 8 September 2023 [64]. Homologs to the MCR proteins and their corresponding gene sequences were then identified by using PATRIC protein family identifiers and corresponding annotations [65,66]. Then all redundant proteins, truncated, low-quality, or distantly related sequences were removed from the analysis. Protein and gene sequences were aligned using MAFFT v7.130b [67]. Alignments were manually curated using JalView V2.11.5.1 [68]. Phylogenetic trees were constructed using FastTree v2.1, employing the Whelan-And-Goldman model for protein evolution [65]. Final trees were visualized using iTOL V7.6 [66].

### 4.6. Data Visualization Using R

All heatmap visualizations were created in R (v4.3.0) using the tidyverse, ComplexHeatmap, circlize, and ggplot2 packages [69]. Binary matrices representing gene presence/absence (e.g., resistance genes and plasmid replicons) were visualized using the ComplexHeatmap package with customized color schemes and annotations. Row and column groupings were handled using the row_split and rowAnnotation functions. Final figures were exported as high-resolution TIFF images at 300 dpi for publication.

## 5. Conclusions

In conclusion, this study provides genomic insights into *Salmonella enterica* isolates from poultry carrying the *mcr-1* gene. The presence of this plasmid-mediated colistin resistance determinant, together with resistance to multiple antimicrobial classes and its occurrence in two epidemiologically important serovars (*S. Infantis* and *S. Minnesota*), highlights the growing threat of antimicrobial resistance within the food chain and its potential for horizontal dissemination. The identification of resistance-associated plasmids, including IncI1 and IncFIB, further underscores the role of mobile genetic elements in the spread of resistance genes. In addition, the observed resistance to commonly used antibiotics such as third-generation cephalosporins, quinolones, and trimethoprim/sulfamethoxazole underscores the potential challenges for conventional treatment options and reinforces the need for continued surveillance and control strategies in poultry production systems.

## Figures and Tables

**Table 1:** The table represents the interpretation of antibiotic susceptibility testing results based on minimum inhibitory concentration (MIC) values. Each result is classified using the following abbreviations: S (Sensitive): The microorganism is susceptible to the antibiotic; R (Resistant): The microorganism is resistant to the antibiotic; and the I (Intermediate): The microorganism has an intermediate response to the antibiotic.

Antibiotic	SA49317 (*S. Minnesota*)	SA49319 (*S. Minnesota*)	SA43930 (*S. Infantis*)	SA43968 (*S. Infantis*)
Amoxicillin–Calvunate	S	S	S	S
Ampicillin	R	R	R	R
Ceftiofur	R	S	R	R
Cefoxitin	R	R	R	R
Cefotaxime	R	S	R	R
Ceftazidime	I	S	S	I
Ceftriaxone	R	S	R	R
Cefepime	S	S	S	S
Colistin	R	R	R	R
Polymixin B	R	R	R	R
Ciprofloxacin	I	I	I	R
Azithromycin	R	R	S	S
Nalidixic Acid	S	S	R	R
Chloramphenicol	R	S	R	R
Tetracycline	R	R	S	R
Tigecycline	S	S	S	S
Amikacin	R	R	R	R
Streptomycin	R	R	R	R
Gentamycin	R	R	R	R
Sulfisoxazole	R	R	S	R
Trimethoprim–Sulfamethoxazole	R	S	R	R
Imipenem	S	S	S	S
Meropenem	S	S	S	S
Ertapenem	S	S	S	S
Temocillin	R	S	R	R
Azteronam	R	R	R	R
Cefotaxime/clavulanic acid	Negative ESBL ^1^	Negative ESBL ^1^	Negative ESBL ^1^	Negative ESBL ^1^
Ceftazidime/clavulanic acid				

^1^ ESBL: Extended-Spectrum β-lactamases.

**Table 2:** The table shows the resistance category of each Salmonella isolate according to the classification criteria established by CLSI ^1^, EUCAST ^2^, and FDA ^3^.

	SA49317 (*S. Minnesota*)	SA49319 (*S. Minnesota*)	SA43930 (*S. Infantis*)	SA43968 (*S. Infantis*)
Total Resistance	17	11	15	18
% of Resistance	65	42	58	69
MAR	0.7	0.4	0.6	0.7
Resistance degree	MDR	MDR	MDR	MDR

^1^ CLSI: Clinical and Laboratory Standards Institute, ^2^ EUCAST: European Committee on Antimicrobial Susceptibility Testing, and ^3^ FDA: Food and Drug Administration.

## Data Availability

All sequence data were deposited in NCBI GenBank under BioProject accession numbers PRJNA844381, PRJNA844392, and PRJNA1398948. The raw reads have been deposited at the Sequence Read Archive (SRA) under accession numbers SRR19759901, SRR19759701, and SRP660113.
